# Synthesis, Characterization, Antimicrobial, Antioxidant, and Anticancer Evaluation of Bio‐Fabricated Silver Nanoparticles (AgNPs) Through *Malus domestica* Peel Extract

**DOI:** 10.1002/cbdv.71913

**Published:** 2026-11-09

**Authors:** Ganesan Rajkumar, Elamaran Anuja, Giriraj Kalaiarasi, Selvaraj Ahalya

**Affiliations:** ^1^ Postgraduate and Research Department of Chemistry Vivekananda College Madurai Tamil Nadu India; ^2^ Department of Chemistry University College of Engineering Anna University BIT Campus Tiruchirapalli Tamil Nadu India; ^3^ Centre for Material Chemistry and Department of Chemistry Karpagam Academy of Higher Education (Deemed to Be University) Coimbatore Tamil Nadu India; ^4^ Postgraduate and Research Department of Chemistry Seethalakshmi Ramaswami College (Affiliated to Bharathidasan University) Tiruchirapalli Tamil Nadu India

**Keywords:** AgNPs, antimicrobial activities, apple peel, biogenic synthesis, *Malus domestica*

## Abstract

The current study involves a greener synthesis of silver nanoparticles (AgNPs) using *Malus domestica* (apple) peel extract. The successful formation of Md‐AgNPs was established by UV–vis spectroscopy, XRD and EDX. The presence of major phytochemicals was evidenced by HPLC analysis. The extract exhibited the total phenolic and flavonoid contents of 198.6 ± 4.8 mg GAE/g dried peel and 82.4 ± 2.3 mg QE/g dried peel, respectively. The Zeta potential of −21.6 mV indicated the stability of the formed Md‐AgNPs. The spherical and monodispersed nature of AgNPs was evidenced by HRTEM and SEM images. The particle size of Md‐AgNPs ranged from 20 to 43 nm, with an average size of 29.9 nm. The Md‐AgNPs demonstrated the concentration‐dependent antibacterial activity, with a maximum zone of inhibition of 13.50 ± 0.70 mm against *Aeromonas hydrophila*. The measured IC_50_ values for Md‐AgNPs using the DPPH and ABTS assays were 100.54 ± 0.28 µg/mL and 194.4 ± 0.19 µg/mL, respectively. The EC_50_ for Md‐AgNPs value determined using FRAP assay was 35.07 ± 0.04 µg/mL. Further, the anticancer activity of the Md‐AgNPs was evaluated against human breast cancer cells and Md‐AgNPs showed potent efficacy (IC_50_ value = 24.8 ± 0.5 µg/mL).

## Graphical abstract

A greener biosynthesis of silver nanoparticles (AgNPs) has been achieved using *Malus domestica* (apple) peel extract. HPLC profile showed the presence of polyphenolic compounds in the apple peel extract. AgNPs were characterized using UV–vis, FTIR, XRD, DLS, Zeta potential, EDX, and elemental mapping analysis. The spherical and monodispersed nature of AgNPs were evidenced from SEM and HRTEM image with an average particle size of 29.9 nm. AgNPs had showed excellent antibacterial, antioxidant, and anticancer activities.

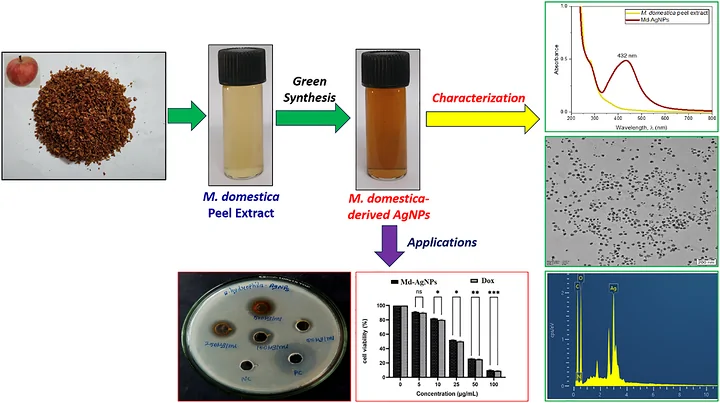

## 1 Introduction

Nanoscience stands out as a vibrant domain within material science due to its interdisciplinary nature. This expansive field encompasses a diverse range of scientific disciplines and involves the manipulation of clusters of atoms, typically ranging from 1 to 100 nm in size [1]. Nanomaterials have received considerable attention due to their distinct characteristics and wide‐ranging applications, particularly in biomedical applications and environmental remediation [2].

Silver nanoparticles (AgNPs) stand out among other metal nanoparticles due to their ease of synthesis and versatility of use. Additionally, AgNPs exhibit remarkable efficacy, chemical stability, and along with numerous beneficial properties including antibacterial, antiviral, antifungal, antioxidant activities, antibiofilm activity, and cytotoxic activities [3]. Conventional chemical methods for synthesizing metal nanoparticles pose environmental risks due to the use of potentially harmful chemicals. Consequently, the greener production of AgNPs has garnered significant interest owing to its eco‐friendly nature, cost‐effectiveness, non‐pathogenic properties and ecological benefits [4, 5].

Polysaccharides derived from plant sources showed potent anti‐inflammatory activities [6, 7]. Biomolecules present in plant extracts, including flavonoids, tannins, phenolics, and proteins, play an important role in the reduction of Ag^+^ to Ag^0^ thereby facilitating the formation of AgNPs [8]. The precise mechanism underlying nanoparticle biosynthesis using different plant extracts remains unclear. These biomolecules participate in the bio‐reduction and capping of the biosynthesized nanomaterials [9]. A diverse array of plant materials has been utilized for nanoparticle synthesis [10, 11].

The greener synthesis of AgNPs using Ajwain leaves has been explored for their therapeutic potential, including antibacterial, anti‐inflammatory, anticancer, and antioxidant effects [12]. Robles‐Martínez et al. reported the antimycotic activity of *Allium sativum* extract and AgNPs against *Trichophyton rubrum* [13]. Relatively few studies have been reported on the synthesis of AgNPs using peel extracts [14, 15]. Typically, fruit peels are discarded after consumption of the pulp. Rasool et al. [16] reported the synthesis of AgNPs using pomegranate fruit peel extract and their application in the degradation of Y‐40 and Red‐227 dyes. Lu et al. reported that the the polyphenols present in the pomace of Gala apple exhibited strong antioxidant activities compared to vitamins C and E [17]. Gorinstein et al. reported that the total polyphenolic contents were higher in the peels of grapefruit, sweet orange and lemon than in their respective pulps [18]. Polyphenols such as catechin and chlorogenic acid was present at higher levels in the peel than in the pulp of peach [19]. Pomegranate peel waste has been utilized for various applications [20]. Although the biogenic production of AgNPs using plant materials has been widely reported, studies utilizing *Malus domestica* (*M. domestica*) peel waste remain very limited.

*M. domestica* is known to contain a several phenolic derivatives [21]. Eberhardt et al. [22] reported significant antioxidant activity in *M. domestica* fruit. Several antioxidant compounds have been reported in both apple flesh and its peels [23]. Israt Jahan et al. [24] described the biogenic synthesis of AgNPs using *M. domestica* pulp. The synthesized AgNPs were effective against pathogenic bacterial strains.

In contrast to earlier *M. domestica*‐based AgNPs synthesis using apple pulp, the current study explicitly explores the apple peel as a resource for AgNPs synthesis. Further, the present study correlates its phenolic composition with the physicochemical characteristics. HPLC analysis, along with total phenolic and flavonoid content determinations, was employed to identify the phytomolecules in the apple peel extract. The bioderived AgNPs were comprehensively analyzed using UV–vis, FT‐IR, XRD, SEM, HRTEM, DLS, and EDX. The antimicrobial activity of *M. domestica*‐derived AgNPs (Md‐AgNPs) was evaluated against pathogenic bacterial strains. Their antioxidant activities were evaluated using the DPPH, ABTS and FRAP method. Further, the anticancer efficacy was evaluated against human breast cancer cells (MCF‐7).

## 2 Methodology

### 2.1 Materials and Chemicals

Silver nitrate (99.9%) was obtained from Merck and used as received. Fresh *M. domestica* fruits were purchased from Sholavanthan market (latitude:10.02383 and longitude:78.22352), Madurai District, Tamil Nadu on 15th January 2026. The fruits were identified and confirmed by Dr. R. Ramasubbu, Associate Professor of Biology, Gandhigram Rural Institute (Deemed to be University), Dindigul, Tamil Nadu (herbarium code: GUD). All aqueous solutions were prepared using deionized water. Prior to use, all glassware and apparatus were carefully washed with aqua regia, rinsed thoroughly with deionized water and thoroughly dried. The bacterial strains *Staphylococcus aureus*‐902, *Escherichia coli*‐443, *Streptococcus pyogenes*‐1928, and *Aeromonas hydrophila*‐1739 were purchased from MTCC, Chandigarh, India. HPLC grade methanol, acetonitrile, formic acid and water were procured from Merck (Darmstadt, Germany). MCF‐7 (human breast cancer cell line) (RRID:CVCL_0031) and human umbilical vein endothelial cells (HUVEC) cells (RRID:CVCL_2959) were obtained from National Centre for Cell Science (NCCS), Pune, India.

### 2.2 Extract Preparation From *M. domestica* Fruit Peel

The fruits of *M. domestica* were washed to remove surface dust particles, and subsequently rinsed thoroughly with deionized water. The fruit skin of *M. domestica* was peeled off and dried in a dark room for about 10 days to obtain a dark brown product (Figure S1). Dried *M. domestica* peel powder was stored in an airtight container for further studies. Exactly 3 g of dried *M. domestica* fruit peel powder (Figure 1a) was heated at 60°C in 50 mL of ultrapure water for 30 min. The resulting extract was filtrated using Whatman No. 1 filter paper.

**FIGURE 1:**
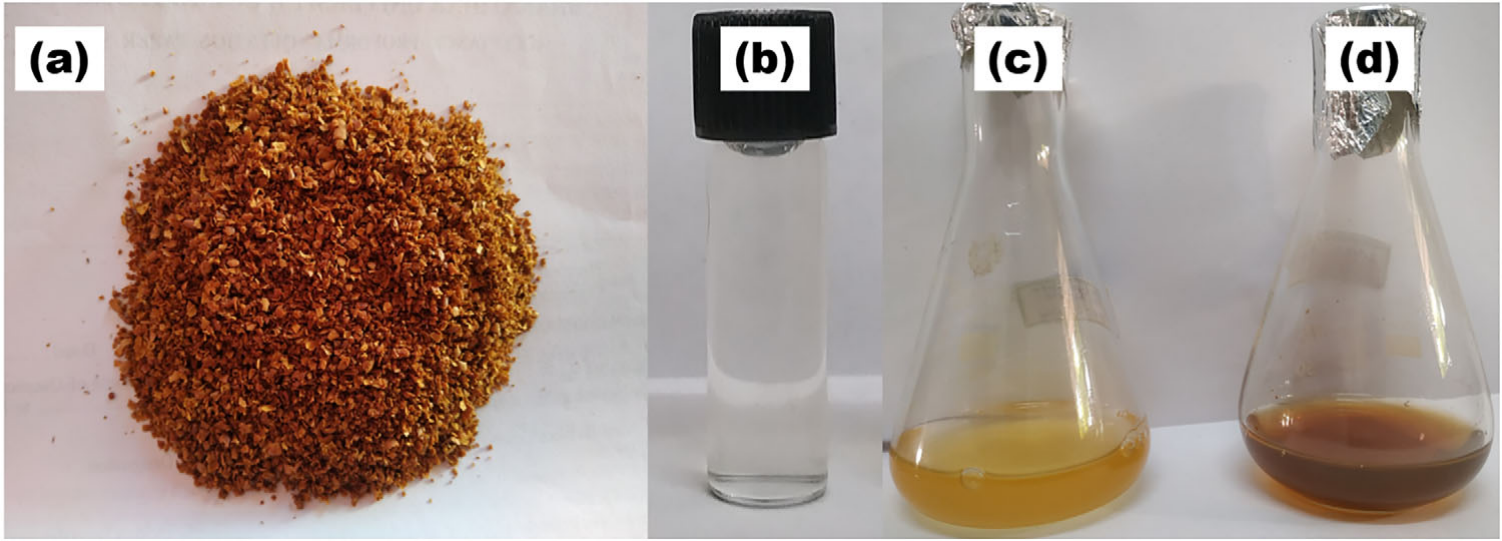
Photographic image of (a) *M. domestica* peel powder; (b) AgNO_3_ solution; (c) *M. domestica* peel extract; (d) Md‐AgNPs.

### 2.3 Preparation of *M. domestica* Peel Extract‐Mediated AgNPs

In a 100 mL Erlenmeyer flask, 3 mL of freshly prepared *M. domestica* peel extract was gradually added to 25 mL of a 1 mM AgNO_3_ solution. The Erlenmeyer flask was placed in a copper water bath at 60°C and observed periodically. After 15 min, the color changed from yellowish to dark reddish‐brown, indicating the production of AgNPs. The colloidal AgNPs were centrifuged at 15 000 rpm for 30 min and the resulting pellet was washed three times with deionized water. The washed pellet was subsequently placed in a hot‐air oven at 120°C for 3 h for drying process. The recovered mass of green‐synthesized Md‐AgNPs was 0.0054 g.

### 2.4 Characterization of Md‐AgNPs

The absorption spectra were recorded using JASCO‐V530 UV–vis spectrophotometer over the wavelength range of 200–800 nm. The FTIR spectrum was recorded using a PerkinElmer spectrometer from 4000 to 400 cm^−^^1^. The particle size was analyzed using a dynamic light scattering (DLS) analyzer Microtrac Inc., (Nanotrac Wave II). High‐performance liquid chromatography (HPLC) analysis was performed using a JASCO HSS 2000i pump, SDM 20ACPDA and AS 2071‐iL autosampler to identify the major phytoconstituents present in the apple peel extract. X‐ray diffraction (XRD) pattern was recorded using a Shimadzu 6000‐X‐Ray Diffractometer using CuKα radiation (*λ* = 1.54056 Å). Scanning electron microscopy (SEM) images were obtained from JEOL Model 6390 computer‐controlled microscope equipped with EDX. The morphology was observed using high‐resolution transmission electron microscopy (HRTEM) image (JEM‐2100 F, JEOL, Japan) operated at 120 kV.

### 2.5 Total Phenolic and Flavonoid Content of *M. domestica* Peel Extract

The total phenolic content (TPC) of the apple peel extract was evaluated using the Folin–Ciocalteu method, with gallic acid as the standard. The obtained results were reported as milligrams of gallic acid equivalents (GAE) per gram of dried peel (mg GAE/g dried peel) and calculated from the corresponding standard calibration curve [25]. The total flavonoid content (TFC) of the apple peel extract was estimated using the AlCl_3_ colorimetric method, with quercetin as the reference standard. The flavonoid content was expressed as milligrams of quercetin equivalents (QE) per gram of dried peel (mg QE/g dried peel) [26]. These contents were determined based on the initial dried *M. domestica* peel powder rather than the dry mass of the aqueous extract. No separate dry‐residue mass or extraction yield of the aqueous peel extract was determined. The measurements such as total phenolic content and flavonoid content obtained from the independent dried *M. domestica* peel powder not the repeated measurements of a single *M. domestica* peel material extract.

### 2.6 High‐Performance Liquid Chromatography (HPLC) Analysis

The phytochemical constituents in the *M. domestica* peel extract were quantitatively analyzed by HPLC technique. HPLC analysis was carried out with a Shimadzu HPLC system equipped with a PDA detector and a C18 column (250 × 4.6 mm, 5 µm) at 25°C. The dried apple peel powder was accurately weighed in glass‐tube, to which analytical grade HPLC methanol was added till a known volume was achieved, sonication for complete dissolving was performed, followed by passing through 0.22 mylon membrane filter. The mobile phase was methanol and 0.1% aqueous formic acid (70:30, v/v) under isocratic conditions at 1.0 mL/min flow rate. The filtered mobile phase was injected in an analytical column (250 × 4.6 mm, 5 µm) in a HPLC‐PDA system and AS 2071‐iL Autosampler. The injection volume was 20 µL and the run time was kept at 10 min. The target compound was quantified at 254 nm and retention time was recorded for identification and quantitative analysis. Chromatograms were printed out and analyzed by using the instrument software. The identity of phytochemical constituent was confirmed by their retention time and absorption spectrum compared with reference compound availability. 10 mg of reference standard was dissolved in 10 mL methanol to get mg/mL (1000 µg/mL) and used as standard stock solution. Working standards at 10, 20, 40, 60, 80, and 100 µg/mL were prepared by serial dilution and each concentration was injected in triplicate. The calibration curve was plotted between mean peak area and standard concentration and found to be linear with *R* ^2^ value. External calibration methods were used for the identification and quantitative assay of phytochemical standards. The corresponding quantitative values were determined by applying a calibration curve, obtained from external standard analysis. Recovery studies were carried out at 80%, 100%, and 120% levels to assess the accuracy and intra‐day and inter‐day precision were determined by three replicate preparations and expressed as % RSD. LOD and LOQ were calculated from the standard deviation of the response and the calibration‐curve. For Table 3, three independent sample preparations were analyzed with triplicate injections and the values reported are mean ± SD (*n* = 3 independent preparations) calculated using the validated calibration equation.

### 2.7 Biological Activities of Md‐AgNPs

#### 2.7.1 Antibacterial Assay

The antibacterial activity of the synthesized Md‐AgNPs (50, 100, 250, and 500 µg/mL) against *Staphylococcus aureus*, *Streptococcus pyogenes*, *Aeromonas hydrophila*, and *Escherichia coli* was assessed by agar well‐diffusion methodology along with the gentamicin (100 µg/mL), AgNO_3_ (100 µg/mL) and *M. domestica* peel dried powder (100 µg/mL). The experimental methodology and statistical analysis are given in the Supporting Information.

#### 2.7.2 Antioxidant Evaluation by DPPH, ABTS, and FRAP Assays

The antioxidant efficacy of the green‐synthesized Md‐AgNPs and the standard (ascorbic acid) was assessed using ABTS•+, DPPH•, and FRAP assays [27, 28, 29, 30, 31]. The detailed experimental procedures, along with the statistical analysis methods, are described in the Supporting Information.

#### 2.7.3 MTT Assay and Cell Morphology Analysis

The anticancer activity of the synthesized Md‐AgNPs and doxorubicin (Dox) was evaluated using the MTT (3‐(4,5‐dimethylthiazol‐2‐yl‐)‐2,5‐diphenyltetrazoliumbromide) assay against MCF‐7 (human breast cancer) cells at various concentrations (5, 10, 25, 50, and 100 µg/mL) for 24 h. Cell morphology analysis of MCF‐7 in the presence of Md‐AgNPs was performed according to the previously described method [32].

## 3 Results and Discussion

### 3.1 Visual Confirmation of Md‐AgNPs

Figure 1 shows the digital photographs of (a) *M. domestica* peel powder; (b) AgNO_3_ solution; (c) *M. domestica* peel extract; (d) Md‐AgNPs. The synthesis of Md‐AgNPs was achieved through the reaction between AgNO_3_ solution and *M. domestica* peel extract. The color changes from yellowish to dark reddish‐brown (Figure 1d) confirmed the successful synthesis of Md‐AgNPs. This color transition was consistent with the previous report by Ibrahim [33].

### 3.2 Total Phenolic and Flavonoid Content of *M. domestica* Peel Extract

The TPC and TFC contents of apple peel extract are presented in Tables 1 and 2, respectively. The TPC of the apple peel extract was found to be 198.6 ± 4.8 mg GAE/g dried peel. As shown in Table 2, the apple peel extract exhibited a TFC of 82.4 ± 2.3 mg QE/g dried peel. The results clearly confirmed that the apple peel extract possess a considerable amount of phenolic and flavonoid compounds. These phenolic and flavonoid compounds may contribute for the stabilization and capping of AgNPs as well as for other biological activities.

**TABLE 1:** Total phenolic content (TPC) of apple peel extract.

Parameters	Values
Total phenolic content	198.6 ± 4.8 mg GAE/g dried peel
Standard	Gallic acid
Unit	mg GAE/g dried peel
Method	Folin–Ciocalteu assay

**TABLE 2:** Total flavonoid content (TFC) of apple peel extract.

Parameters	Values
Total flavonoid content	82.4 ± 2.3 mg QE/g dried peel
Standard	Quercetin
Unit	mg QE/g dried peel
Method	Aluminum chloride colorimetric assay

### 3.3 HPLC Analysis of *M. domestica* Peel Extract

The phytochemical constituents and their composition in the extract directly impact the reduction of Ag^+^ into AgNPs. The phytochemicals present in *M. domestica* peel extract were confirmed by HPLC analysis and are presented in Figure S2 and Table 3. The *M. domestica* peel extract contains phytochemicals including gallic acid, catechin, chlorogenic acid, epicatechin, rutin, hyperoside, phloridzin, and quercetin. A total of eight phenolic compounds were identified, with phloridzin being the major biomolecule in the extract (26.4%). Epicatechin was the second most abundant biomolecule (16.38%), followed by chlorogenic acid (16.3%).

**TABLE 3:** HPLC analysis of *M. domestica* peel extract.

Peak nos.	Compounds identified	Retention time (min)	Peak area (mAU·s)	Peak height (mAU)	Concentration (mg/g extract)	Relative area (%)
1.	Gallic acid	3.18	165 423	8 945	1.42 ± 0.05	1.94
2.	Catechin	5.67	945 876	56 241	8.96 ± 0.32	11.11
3.	Chlorogenic acid	7.34	1 387 542	81 456	13.74 ± 0.41	16.29
4.	Epicatechin	9.28	1 643 254	96 542	16.38 ± 0.52	19.29
5.	Rutin	12.86	615 482	35 416	5.81 ± 0.21	7.23
6.	Hyperoside	15.73	1 015 467	61 574	9.54 ± 0.36	11.92
7.	Phloridzin	18.92	2 245 675	128 945	21.48 ± 0.65	26.37
8.	Quercetin	22.65	503 246	30 241	4.76 ± 0.18	5.91

### 3.4 UV–Vis Spectral Analysis

Figure 2a illustrates the UV–vis spectra of *M. domestica* peel extract and Md‐AgNPs. In UV–vis spectroscopy, metallic nanoparticles exhibit characteristic absorption bands within specific ranges of the electromagnetic spectrum. AgNPs typically manifest a distinctive sharp surface plasmon resonance (SPR) band in the range of 400–475 nm [34]. The SPR band of the biosynthesized Md‐AgNPs was observed at 432 nm. Similar observations were reported by Musere et al. [35]. The SPR band for AgNPs derived from the aerial parts of *Erythrina abyssinica* was also in agreement with our findings [36]. A diverse array of phytochemicals presents in the extract played a crucial role in the formation and stabilization of nanoparticles [37]. The phytoconstituents present in the peel extract are likely to contribute to the reduction of Ag^+^ ions to Ag^0^. The biomolecules identified by HPLC method possess reducing properties and effectively reduced Ag^+^ ions into AgNPs [38].

**FIGURE 2:**
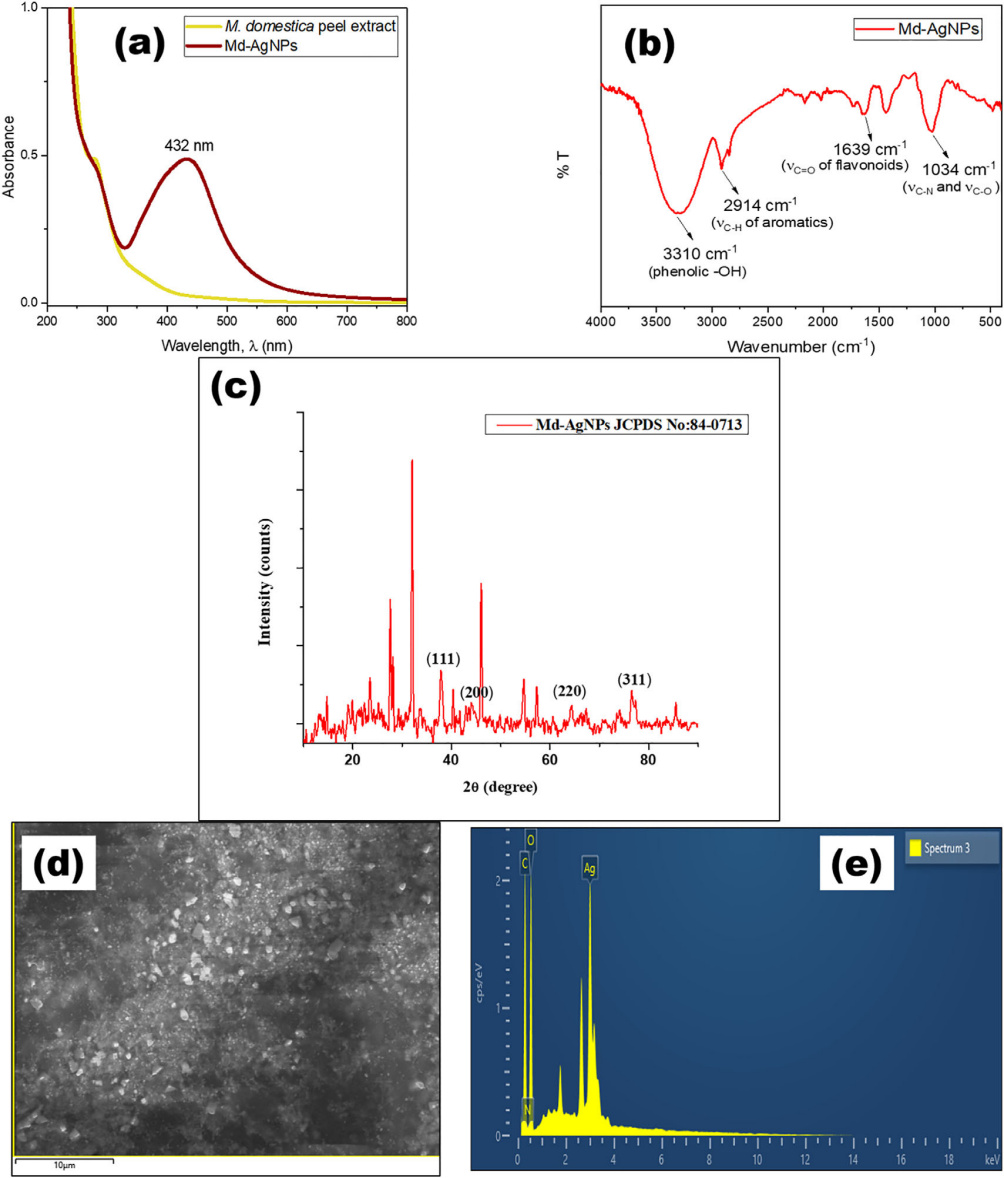
(a) UV–vis spectrum; (b) FT‐IR spectrum; (c) XRD; (d) SEM; (e) EDX spectra of Md‐AgNPs.

### 3.5 FTIR Analysis

FTIR analysis was employed to recognize the biomolecules responsible for the formation of AgNPs. Figure 2b depicts the FTIR spectrum of Md‐AgNPs. The Md‐AgNPs showed stretching frequency values at 3310, 2914, 1639, and 1034 cm^−1^. The peak observed at 3310 cm^−1^ could be due to the presence of O─H bonds associated with alcohols/phenolic derivatives. The band at 2914 cm^−1^ is due to the ‐OH stretching of carboxylic acids and the C─H stretching of aromatic derivatives [39]. The stretching frequency value at 1034 cm^−1^ is attributed to the *ν*_C─N_ and *ν*_C─O_ of amino and carboxylic acid groups, mainly originating from flavonoids and acids present in *M. domestica* peel extract. Furthermore, the stretching band at 1639 cm^−1^ could be due to the C═O group of aldehyde or ketone functional groups. These observations indicate that the phytomolecules in the *M. domestica* peel extract played an important role in reducing Ag^+^ to AgNPs (Ag^0^), as well as in capping and stabilizing the nanoparticles. The present findings are in agreement with earlier report on the biogenic synthesis of AgNPs [40].

### 3.6 PXRD Analysis

XRD analysis revealed the crystalline parameters of the biogenically synthesized Md‐AgNPs as shown in Figure 2c. The XRD spectrum revealed diffraction peaks at 2*θ*  = 38.08°, 44.15°, 64.45°, 77.32° which correspond to face‐centered cubic metallic Ag nanoparticles (JCPDS no. 84‐0713) [41]. In addition, additional peaks at approximately 32.00°, 46.11° and 54.49° were also observed, which could be attributed to residual biomolecules capped on the surface of the AgNPs [42]. The average crystallite size (*D*) of the biogenically synthesized Md‐AgNPs was calculated using the Debye‐Scherrer equation and was found to be 24.05 nm. Dislocation density (*δ*) is defined as the number of dislocations per unit volume and was calculated using equation given below. The dislocation density (*δ*) of the biogenically synthesized Md‐AgNPs was found to be 1.72 × 10^−3^ nm^−2^.

$$\delta =\frac{1}{D^{2}}$$

The average crystallite size (*D*) of the biogenically synthesized Md‐AgNPs was calculated using the Debye–Scherrer equation and was found to be 24.05 nm. Dislocation density (*δ*) is defined as the number of dislocations per unit volume and was calculated using the equation given below. The dislocation density (*δ*) of the biogenically synthesized Md‐AgNPs was found to be 1.72 × 10^−^^3^ nm^−^^2^.

Microstrain (*ε*) generally occurs in the solids due to the presence of a higher proportion of surface atoms and unsaturated bonds. The micro strain of the biogenically synthesized Md‐AgNPs was determined to be 7.00 × 10^−^^3^. The microstrain was calculated using the following equation:

$$\varepsilon =\frac{\beta }{4\tan\theta }$$

### 3.7 SEM and EDX

The SEM image of bio‐fabricated AgNPs is shown in Figure 2d. The SEM image revealed that the Md‐AgNPs exhibited a crystalline morphology, with a homogeneous and regular shape. The particles exhibited different sizes, which may be attributed to agglomeration. This agglomeration could be due to the nucleation effects or surface energy interactions [43].

The EDX pattern of Md‐AgNPs is shown in Figure 2e and the percentage composition is presented in Table 4. The EDX spectrum clearly demonstrated the presence of Ag in the AgNPs with a characteristic signal at 3 keV. The signals for oxygen and carbon observed in the EDX spectrum could be attributed to the phytomolecules present on the surface of the Md‐AgNPs. The observed results are consistent with the previous findings of Mahadevan et al. [44].

**TABLE 4:** Elemental composition of biogenically synthesized Md‐AgNPs.

Elements	Line types	Wt%	Wt% sigma	Atomic%
Ag	L series	35.48	0.70	6.68
O	K series	36.46	0.72	46.29
C	K series	26.29	0.60	44.46
N	K series	1.77	1.27	2.56

The real time distribution of the elements in the Md‐AgNPs was also recorded using EDX coupled with electron microscopy. The elemental mapping images of the Md‐AgNPs are presented in Figure 3a–e. The images show the presence and distribution of Ag in the synthesized Md‐AgNPs (Figure 3a). In addition to the strong silver signal, the elemental mapping also revealed the presence of carbon (C), oxygen (O), and nitrogen (N) in the Md‐AgNPs (Figure 3b–e).

**FIGURE 3:**
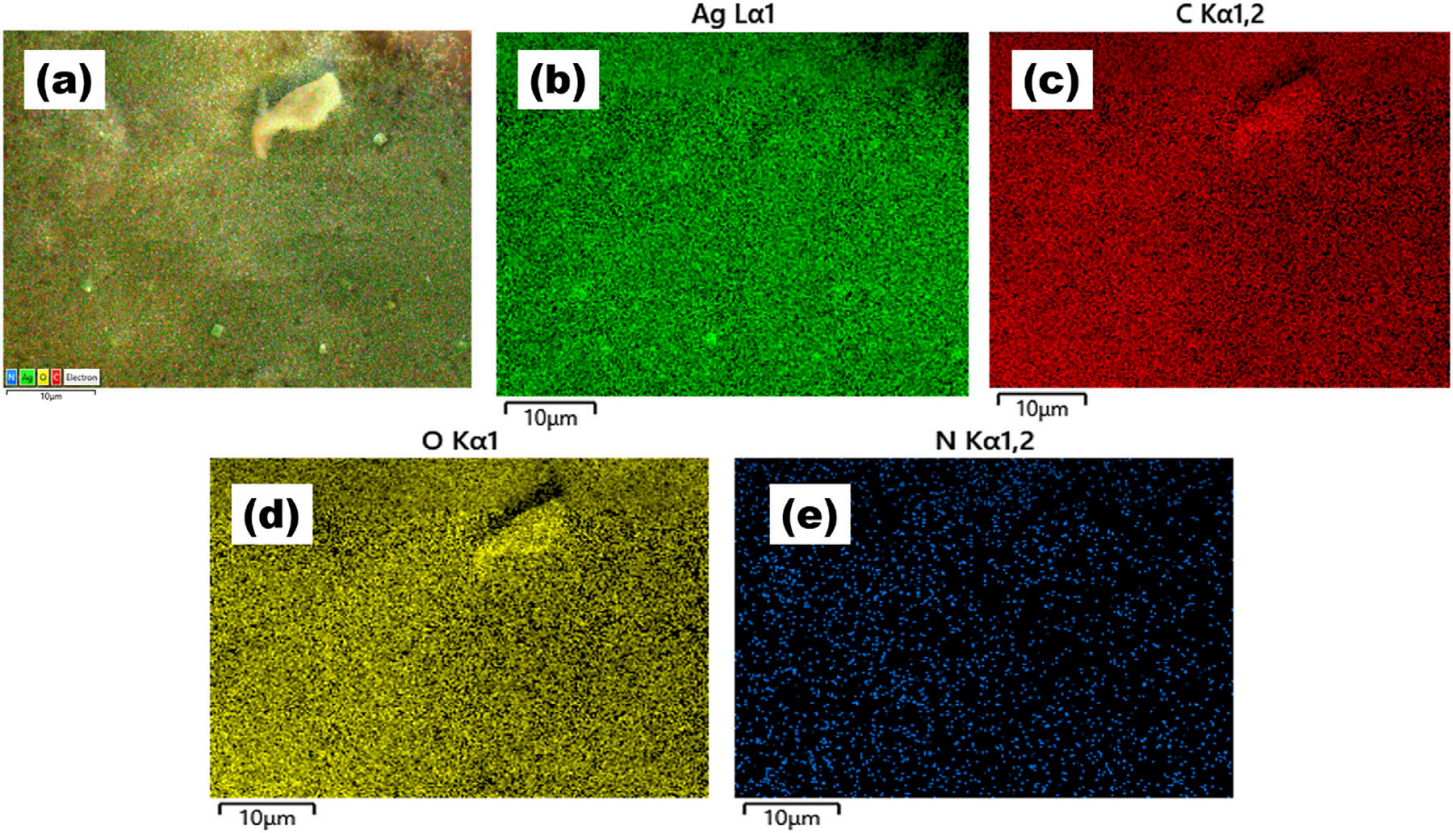
(a) X‐ray mapping of Md‐AgNPs. (b) X‐ray mapping of silver; (c) carbon; (d) oxygen, and (e) nitrogen in Md‐AgNPs.

### 3.8 DLS and Zeta Potential Measurement

The aggregation of nanoparticles depends on the electrical characteristics and surface charge, which influence the interactions between particles. Consequently, a Zeta potential value of ±30 mV suggests the stability of metal nanoparticles, whereas a value between ±10 is typically unstable [45]. In the present study, the value was determined to be −21.6 mV, indicating moderate stability of Md‐AgNPs. Similar Zeta potential value (−24.6 mV) was reported for the AgNPs synthesized using green tea extract [46]. The polydispersity index (PDI) serves as a measure of nanoparticle size distribution. In the present work, the PDI of the green‐synthesized Md‐AgNPs was determined to be 0.1610, suggesting that the particles were monodispersed [47]. The DLS analysis revealed an average hydrodynamic diameter of 33 nm for the Md‐AgNPs.

### 3.9 HRTEM

The morphological characteristics of Md‐AgNPs were examined using HRTEM image and the image is shown in Figure 4a. As evident from the HRTEM image, the biosynthesized Md‐AgNPs exhibited a spherical shape and were evenly distributed. Similar observations were reported by Jyoti et al. [48]. This indicated the stability of the bioinspired AgNPs derived from *M. domestica* peel extract. The particle size histogram (Figure 4b) indicated that the Md‐AgNPs sizes ranging from 20 to 43 nm, with a mean size of 29.9 nm.

**FIGURE 4:**
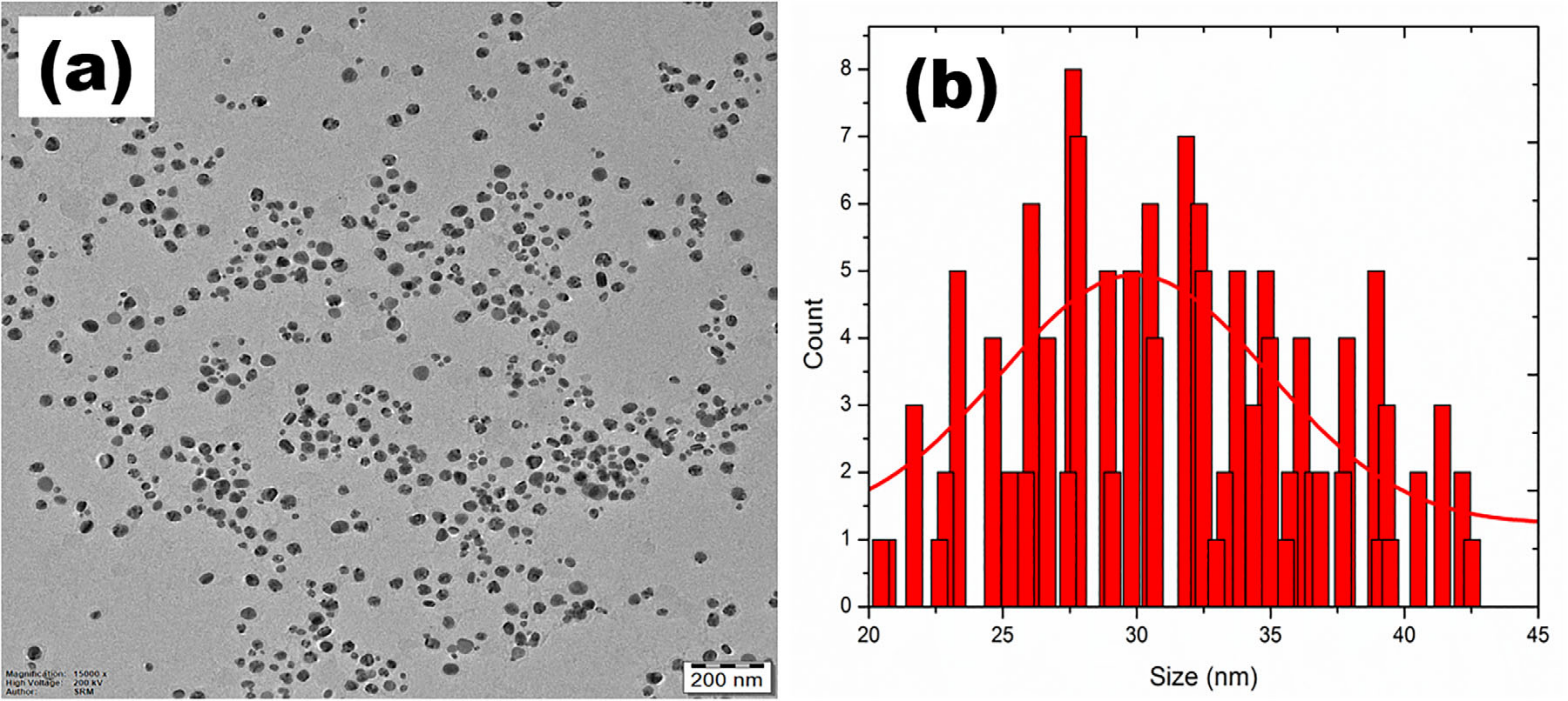
(a) HRTEM image. (b) particle size distribution histogram of Md‐AgNPs.

The particle size of Md‐AgNPs showed slight variation among the different characterization techniques namely XRD, DLS, and HRTEM. The HRTEM revealed an average particle size of 29.9 nm, while the size calculated from XRD was 24.05 nm. The smaller size obtained from XRD can be attributed to the measurement of individual crystalline domains, whereas HRTEM provides the overall physical size of the nanoparticles. The DLS showed an average hydrodynamic diameter of 33 nm, which was slightly higher than the HRTEM size, as DLS measures the particle together with its associated phytochemical capping layer. In addition, differences in the time interval between sample preparation and characterization may also contribute to variations in the measured hydrodynamic size. Similar size variation was reported in the literature for the green synthesized AgNPs [49].

### 3.10 Assessment of Biological Activities of AgNPs

#### 3.10.1 Antibacterial Evaluation of AgNPs

The antibacterial activity of the phytosynthesized Md‐AgNPs, Md‐extract, AgNO_3_, and gentamycin was evaluated against *S. aureus*, *S. pyogenes*, *A. hydrophila*, and *E. coli*. The ZOI was measured at various concentrations of Md‐AgNPs (50, 100, 250, and 500 µg/mL) (Figure S3) are tabulated in Table 5. The antibacterial activity of the Md‐AgNPs was found to be concentration‐dependent. The activity of the Md‐AgNPs was compared with the positive control gentamicin, AgNO_3_ and *M. domestica* peel extract. Md‐AgNPS showed less activity compared to the positive control and shows significant activity than AgNO_3_ and *M. domestica* peel extract (Table 5) at 100 µg/mL. Remarkably, the biosynthesized Md‐AgNPs exhibited antibacterial activity against *A. hydrophila* compared to that of the positive control. No antibacterial activity was observed for the negative control. Further minimum inhibitory concentration (MIC) and minimum bactericidal concentrations (MBC) had been examined and the obtained results are given in the Tables S1 and S2, respectively. The Md‐AgNPS exhibited MICs of 50 µg/mL against *S. aureus*, *S. pyogenes*, and *E. coli* and 25 µg/mL against *A. hydrophila*. The corresponding MBC values were 100 µg/mL for *S. aureus*, *S. pyogenes*, and *E. coli* and 50 µg/mL for *A. hydrophila*. The morphology and other physiochemical characteristics of nanoparticles play pivotal roles in determining their antibacterial potential [50]. AgNPs have been shown to increase cell membrane permeability, leading to protein leakage. Additionally, the generation of reactive oxygen species (ROS) may disrupt the bacterial respiratory chain and inhibit lactate dehydrogenase activity [51].

**TABLE 5:** Zone of inhibition (mm) for the Md‐AgNPs, Md‐extract, AgNO_3_, and gentamicin. The data is presented as mean ± standard deviation. *p*‐Values were calculated using a one‐way ANOVA with Tukey's HSD post‐hoc test and significance levels were denoted as *p* < 0.05. Values are expressed as mean ± SD (*n* = 3). Within each row, means followed by different superscript letters differ significantly (*p* < 0.05).

S. nos.	Name of the bacteria			Zone of inhibition (mm) mean ± SD				
		Positive control (gentamicin) (100 µg/mL)	AgNO_3_ (100 µg/mL)	Md‐extract (100 µg/mL)	Md‐AgNPs			
					50 µg/mL	100 µg/mL	250 µg/mL	500 µg/mL
1.	*S. aureus*	14.50 ± 0.70^a^	7.50 ± 0.49^d^	4.12 ± 0.69^e^	8.25 ± 0.35^c,d^	9.25 ± 0.35^b,c^	9.50 ± 0.70^b,c^	10.50 ± 0.70^b^
2.	*S. pyogenes*	14.25 ± 0.35^a^	6.92 ± 0.36^d^	3.43 ± 0.55^f^	5.25 ± 0.35^e^	8.25 ± 0.35^c^	9.25 ± 0.35^b,c^	10.25 ± 0.35^b^
3.	A. hydrophila	13.50 ± 0.70^a^	8.16 ± 0.70^c^	5.20 ± 0.15^d^	6.25 ± 0.35^d^	10.5 ± 0.70^b^	12.50 ± 0.70^a^	13.50 ± 0.70^a^
4.	E. coli	13.70 ± 0.71^a^	6.14 ± 0.36^c^	6.33 ± 0.19^c^	4.25 ± 0.35^d^	5.25 ± 0.35^c,d^	11.25 ± 0.35^b^	12.25 ± 0.35^b^

#### 3.10.2 Antioxidant Activities of Md‐AgNPs

The antioxidant efficacy of the phyto‐mediated Md‐AgNPs was evaluated using the DPPH, ABTS, and FRAP assays, along with the ascorbic acid (AA) as the standard. A high number of free radicals can induce oxidative stress in cells and cause various diseases. Antioxidants scavenge free radicals by donating electrons to them, thereby converting them into a stable state and neutralizing them [52].

2,2‐Diphenyl‐1‐picrylhydrazyl (DPPH) is a free radical and reduced to DPPH‐H (diphenylpicrylhydrazine) in the presence of an antioxidant. The Md‐AgNPs exhibited good antioxidant activity and with the increasing concentrations of Md‐AgNPs, the percentage of inhibition also increased (Figure 5). IC_50_ value of the Md‐AgNPs and AA were found to be 100.54±28 µg/mL and 7.97±0.31 µg/mL, respectively. The obtained IC_50_ values were compared using Welch's *t*‐test (*t* = 5.73; df = 2; *p* = 0.029), indicating a significantly higher IC_50_ for Md‐AgNPs compared to the AA. The antioxidant activity of the present work is comparable with previous studies reported in the literature [53].

**FIGURE 5:**
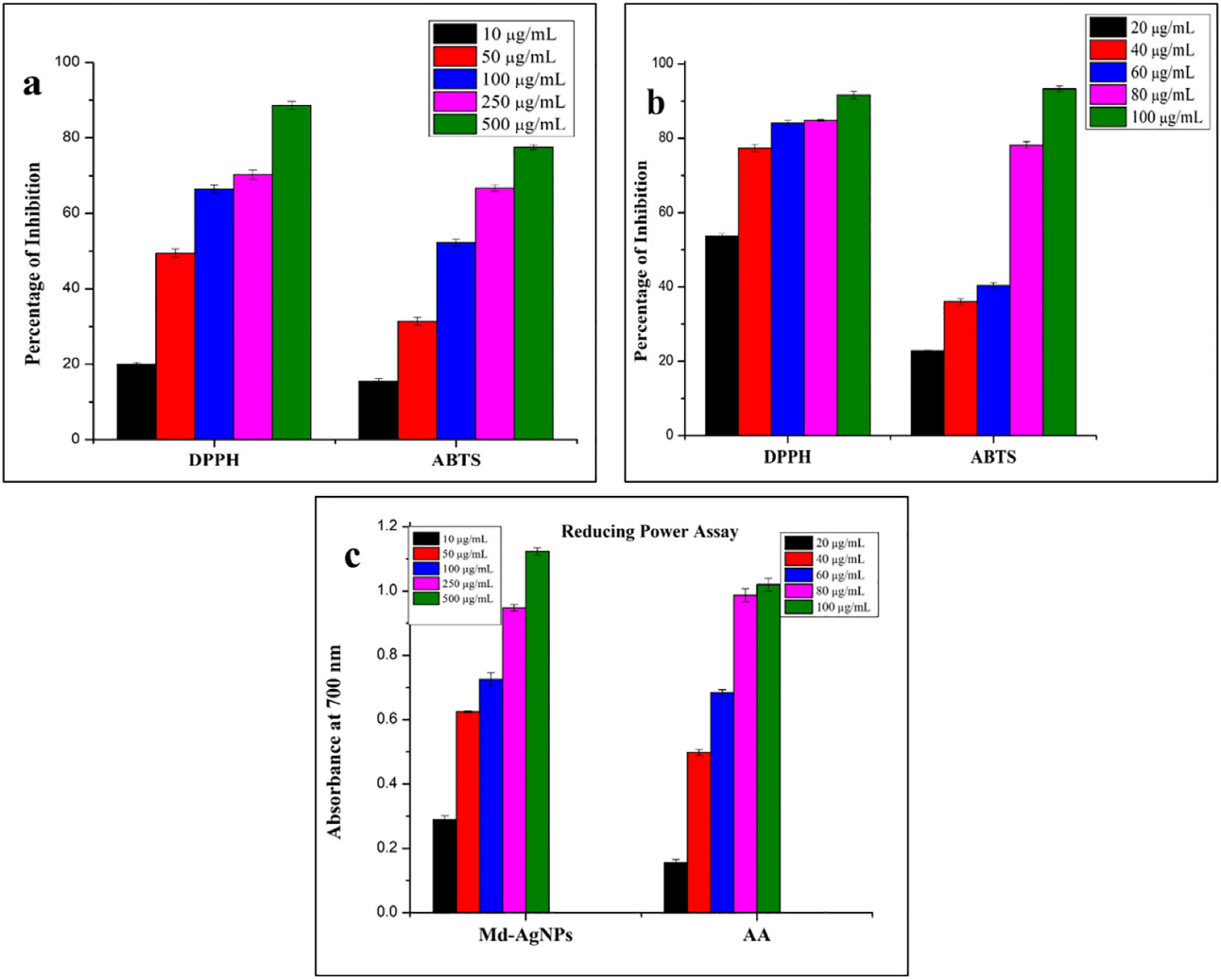
(a) Inhibition of DPPH and ABTS radicals by Md‐AgNPs. (b) Inhibition of DPPH and ABTS radicals by ascorbic acid (AA). (c) Reducing power ability of Md‐AgNPS (10–500 µg/mL) and AA (20–100 µg/mL). Error bars represent the standard deviation of the mean (*n* = 3).

ABTS^+^ (2,2'‐azino‐bis (3‐ethylbenzothiazoline‐6‐sulfonic acid) is a bluish‐green unstable radical. This radical is formed by the oxidation of ABTS with potassium persulfate. The antioxidant activity of the Md‐AgNPs is directly proportional to the degree of decolorization after the addition of compounds [54]. While increasing concentrations of Md‐AgNPs, the percentage of inhibition was also increased (Figure 5). The IC_50_ values of the Md‐AgNPs and AA in ABTS^+.^ free radical scavenging assay were 194.4±19 µg/mL and 55.45±31 µg/mL, respectively. The obtained results were compared using Welch's *t*‐test (*t* = 6.62; df = 3.32; *p* = 0.0051), indicating a significantly higher IC_50_ for Md‐AgNPs compared to the AA. Gecer et al. also reported similar antioxidant effects for AgNPs synthesized using *Echium vulgare* [55].

The FRAP (ferric reduction antioxidant power) assay was employed to evaluate the antioxidant potential of the metal NPs by measuring their ability to convert ferric ions (Fe^3+^) to ferrous ions (Fe^2+^) through electron donation at 700 nm. The EC_50_ values of the Md‐AgNPs and AA in FRAP assay were found to be 35.07±0.04 µg/mL and 40.21±0.05 µg/mL, respectively and the obtained results were compared with Welch's *t*‐test (*t* = 139.04; df = 3.82; *p* = 0.0001). The EC_50_ value obtained for bio‐derived Md‐AgNPs was significantly lower than that of ascorbic acid (AA) and suggested a highly efficient electron‐transfer capability [56]. The results of the study had clearly exposed the antioxidant efficiency of bio‐fabricated AgNPs. This could be due to the presence of polyphenolics capped on the Md‐AgNPs. Flavanols and major polyphenols found in *M. domestica* peel extract contribute to its strong bioactivities [57, 58].

#### 3.10.3 Anticancer Activity of Md‐AgNPs

The anticancer potential of the synthesized Md‐AgNPs was assessed against human breast cancer cells (MCF‐7) using MTT assay (Figure S4), with Dox as the standard, after an incubation period of 24 h. The morphological images of MCF‐7 cells treated with Md‐AgNPs are shown in Figure 6. The green synthesized Md‐AgNPs inhibited the growth and proliferation of MCF‐7 cells, with an IC_50_ value of 24.8±0.5 µg/mL. This activity is comparable with that of the standard drug, Dox (9.7±0.5 µg/mL), against MCF‐7 cells. This result demonstrated the cytotoxic activity of the Md‐AgNPs against MCF‐7 cells. Similar anticancer activity of AgNPs synthesized using *S. macrophylla* leaf extract was reported by Sivalingam [59]. To examine the selectivity of Md‐AgNPs, MTT assay was carried out with human umbilical vein endothelial cells (HUVEC) (Figure S5) and the activity of the Md‐AgNPs was found to be less (IC_50_>200 µM) with HUVEC cells, which revealed that Md‐AgNPs was selective to cancer cells rather than normal cells. The untreated cells (control cells) maintained their morphology and Md‐AgNPs treatment induced cell separation and nuclear shrinkage, accompanied by a reduction in viable cell numbers. The results of the present study clearly demonstrated that Md‐AgNPs exerted a significant cytotoxicity against MCF‐7 cells.

**FIGURE 6:**
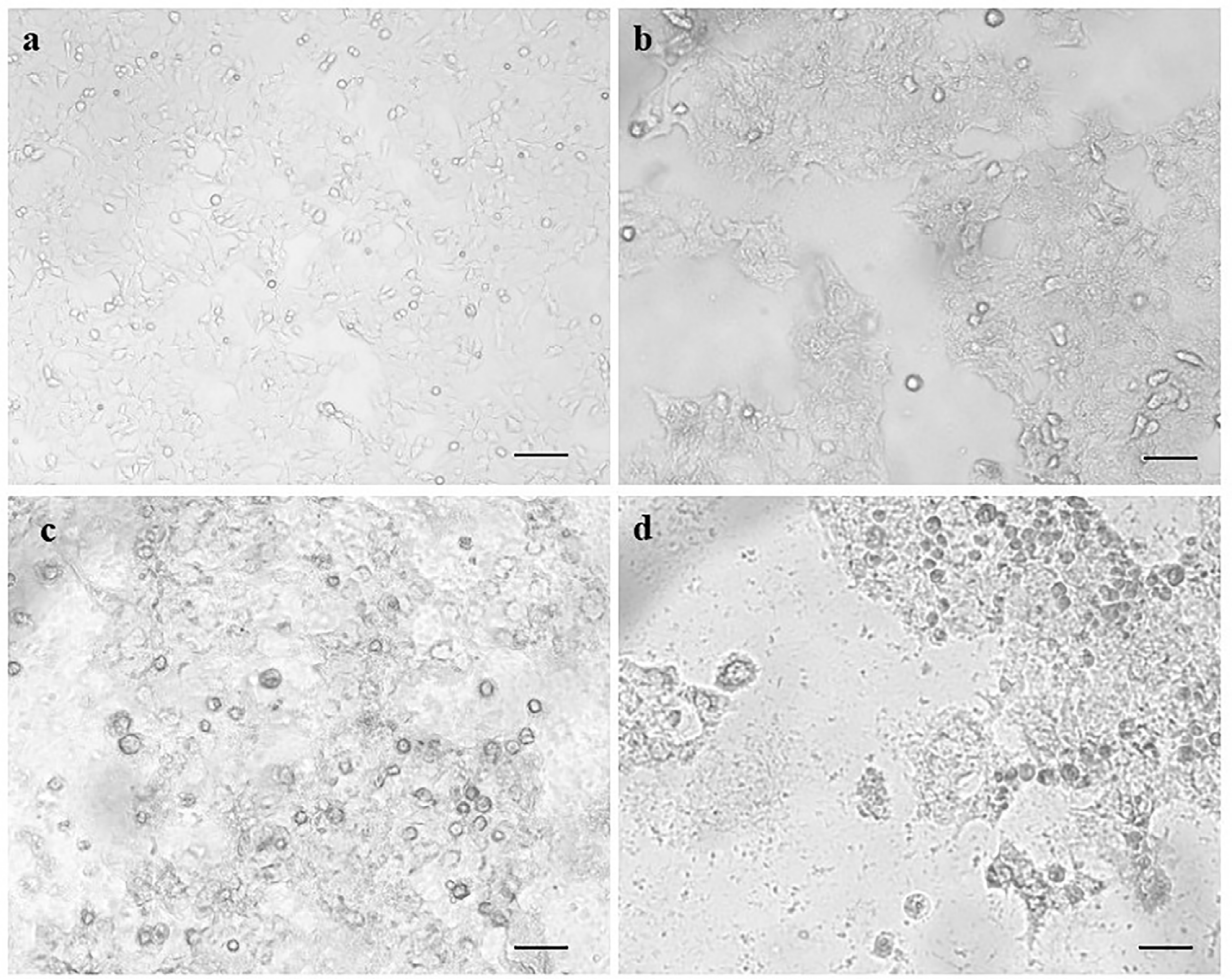
Inverted light microscopic imaging. Effect of Md‐AgNPs on the viability of MCF‐7 cells: (a) Control; (b) 10 µg/mL; (c) 25 µg/mL; (d) 50 µg/mL (scale bar‐50 micrometer).

## 4 Conclusions

The polyphenolic compounds present in *M. domestica* fruit peel were involved in the formation of Md‐AgNPs. HPLC analysis confirmed the presence of polyphenolics in the *M. domestica* peel. The production of Md‐AgNPs was confirmed by the SPR band observed in the UV–vis spectrum. The average size determined using HRTEM was 29.9 nm. XRD, EDX, and elemental mapping confirmed the presence of elemental silver in the biosynthesized Md‐AgNPs. The bio‐fabricated Md‐AgNPs showed a concentration‐dependent antimicrobial effect against the tested pathogens. The results of DPPH, ABTS, and FRAP assays demonstrated the antioxidant efficacy of Md‐AgNPs. This could be due to the polyphenolic compounds capped over the surface of the Md‐AgNPs. Furthermore, in vitro evaluation against MCF‐7 cells revealed concentration‐dependent cytotoxicity of green‐synthesized AgNPs. The present findings provide a preliminary basis for the further research. However, more studies on the mechanism of action, toxicity, biocompatibility, and in vivo effects are needed before their biomedical and pharmaceutical applications. The applications of these biosynthesized AgNPs in food packaging and other field also needs to be investigated in future studies.

## Author Contributions

**Ganesan Rajkumar**: data curation, visualization, conceptualization, funding acquisition, investigation, methodology, formal analysis, software, validation, supervision, writing – review and editing, writing – original draft preparation, and project administration. **Elamaran Anuja**: methodology, software, and resources**. Giriraj Kalaiarasi**: data curation, formal analysis, software, visualization, writing – review and editing, and validation. **Selvaraj Ahalya**: resources and visualization.

## Funding

The authors have nothing to report.

## Ethics Statement

The authors have nothing to report.

## Conflicts of Interest

The authors declare no conflicts of interest.

## Use of Generative AI and AI‐Assisted Technologies in the Writing Process

The authors declare that no generative artificial intelligence or AI‐assisted technologies were used in the research process, data analysis, or manuscript preparation of the present study.

## Supporting information




**Supporting File**: cbdv71913‐sup‐0001‐SuppMat.docx.

## Data Availability

The data that support the findings of this study are available from the corresponding author upon reasonable request.
