# Supplementary material for: Synthesis, Characterization, Antimicrobial, Antioxidant, and Anticancer Evaluation of Bio‐Fabricated Silver Nanoparticles (AgNPs) Through Malus domestica Peel Extract
Source: Chem Biodivers. 2026 Nov 9;23(11):e71913. doi: 10.1002/cbdv.71913 (PMC13749056; doi:10.1002/cbdv.71913)
Supplement: Supplementary file 1 [file CBDV-23-e71913-s001.docx]

**Antibacterial activities**

The antibacterial activity of the synthesized Md-AgNPs against *Staphylococcus aureus, Streptococcus pyogenes, Aeromonas hydrophila* and *Escherichia coli* was evaluated using the agar well-diffusion method [S1]. Nutrient Agar (HiMedia) was used as the agar medium. It was prepared by dissolving 2.8 g of the medium in 100 mL of distilled water. The medium was sterilized by autoclaving at 121 °C and 15 psi for 15 min. After autoclaving, the molten medium was mixed thoroughly and aseptically poured into 100 mm Petri plates at a volume of 20 mL per plate. The bacterial strains were sub-cultured in nutrient agar medium for 24 h. Then, it was adjusted to 0.5 OD according to the McFarland standard before inoculation onto Nutrient Agar plates. Different concentrations of Md-AgNPs (50, 100, 250 and 500 µg/mL) were added to the respective wells and incubated at 37 ^o^C. The zones of inhibition (ZOI) measured to evaluate the antibacterial activity of the Md-AgNPs. Gentamicin (100 µg/mL) was used as the positive control, while AgNO₃ (100 µg/mL) and *M. domestica* peel (100 µg/mL) extract were served as the controls to compare the activity of the Md-AgNPs.

**Sample preparation for MIC and MBC evaluation**

From each sample received, three different concentrations of 25 µg/mL to 100 µg/mL solutions were prepared. Each microbial strain was centrifuged at 11,000 rpm for 5 min in Brain Heart Infusion (BHI) broth. 20 mL of sterile normal saline was added after discarding each microbial supernatant. The remaining individual microbial precipitate were viable and maintained as stock. Then, the concentration of each microbial solution was adjusted by spectrophotometer to an optical density of 0.10 at 625 nm (10^8^ CFU/mL, 0.5 McFarland’s standard).

**MIC determination**

The standard broth dilution method was used to study the antimicrobial efficacy of the samples by evaluating the visible growth of microorganisms in the agar broth. Serial two-fold dilutions of each sample in concentrations ranging from 25 µg/mL to 250 µg/mL with adjusted microbial concentration (10^8^ CFU/mL, 0.5 McFarland’s standard) were used to determine MIC in BHI broth. The control contained only inoculated broth and incubated for 24 h at 37°C. The MIC endpoint is the lowest concentration of the sample where no visible growth is seen in the tubes. The visual turbidity of the tubes was noted, both before and after incubation to confirm the MIC value.

**MBC determination**

After the MIC determination of each sample, aliquots of 50 µL from all the tubes which showed no visible bacterial growth were seeded on BHI agar plates and incubated for 24 h at 37°C. When 99.9% of the microbial population is killed at the lowest concentration of an antimicrobial agent, it is termed as MBC end point. This was done by observing pre and post-incubated agar plates for the presence or absence of bacteria.

**Statistical analysis**

All antibacterial assays were performed in triplicate (n = 3), and Zone of inhibition are expressed as mean ± standard deviation (SD). Statistical comparisons among positive control, AgNO_3_, Md-extract, and Md-AgNPs (50–500 µg/mL) were performed individually for each bacterial strain using one-way analysis of variance (ANOVA), followed by Tukey's post-hoc test. Differences were considered statistically significant at p < 0.05. Statistical analyses were performed using **GraphPad Prism 6.0.**

**Antioxidant assays**

The antioxidant capacities were systematically assessed using three different assays, such as ABTS, DPPH and FRAP assays. Different concentration of stock solutions of the Md-AgNPS (10 µg/mL, 50 µg/mL, 100 µg/mL, 250 µg/mL, 500 µg/mL) were used along with the standard (Ascorbic acid, AA) (20-100 µg/mL).

**DPPH scavenging activity**

The potential of 1,1-diphenyl-2-picrylhydrazyl (DPPH) free radical scavenging activity was measured using the method. Different concentrations of compounds (10 µg/mL,50 µg/mL, 100 µg/mL, 250 µg/mL,500 µg/mL) and standard were added to 5 mL of 0.1 mM methanolic solution of DPPH [S2]. The mixture was shaken followed by inhibiting at room temperature for 30 min in dark. The absorbance against blank was measured at 517 nm.

**ABTS ^.+^ scavenging activity**

The total antioxidant capacity of the compounds was determined by the
2,2′-azinobis-(3-ethylbenzothiazoline-6-sulphonic acid diammonium salt (ABTS) radical cation decolourisation assay [S3]. In brief, the radical cation ABTS^.+^ generated by a reaction between ABTS (7 mM) and persulphate (2.45 mM) was kept for incubation in dark at room temperature about 12-16 h. Then the mixture solution was diluted with ethanol (1:89) to give an ABTS^.+^ solution with the absorbance reaching 0.70 ± 0.02 at 734 nm. Then Md-AgNPS (10-500 μL) and AA (20-100 μL) of the stock solution was added to 2 mL of the ABTS^.+^ solution, kept in the dark at room temperature for 10 min and the absorbance was measured against blank.

All readings were taken in triplicate at each concentration to generate consistent data with statistical errors. The % inhibition was calculated by following equation.

$\% of inhibition=\frac{A_{0}-A_{c}}{A_{0}} \times100$	(1)

*A_o_* and *A_c_* are the absorbance in the absence and presence of the tested compounds, respectively. The percentage of radical inhibition was plotted against compound concentration (µg/mL), and linear regression was applied to the linear portion of the curve (Y = mX + c). IC₅₀, the concentration required for 50% inhibition, was calculated as IC₅₀ = (50 − c)/m.

**FRAP (Ferric reducing antioxidant power) assay**

The reducing power of Md-AgNPS and AA was determined by the ferric reducing antioxidant power (FRAP) method prescribed by Benzie and Strain [S4]. The FRAP solution was prepared by mixing 2.5 mL of 10 mM 4,6-tripyridyl-s-triazine prepared in 40 mM HCl, 2.5 mL of 20 mM FeCl_3_, 25 mL of acetate buffer (pH ~ 3.6), and 3 mL of distilled water. About 30 µL of the different concentrations of the sample was mixed with 970 µL of FRAP solution and incubated for 30 min at 37 °C. The reaction was monitored using UV–Vis spectroscopy by measuring the absorbance at 593 nm. The EC_50_ defined as the sample concentration required to produce an absorbance of 0.5 which was calculated from the plot of absorbance against sample concentration [S5]. EC_50_ was calculated from the linear regression equation (Abs = m·C + c) by solving for the concentration at Abs = 0.5.

**Statistical analysis**

All assays were performed in triplicate (n = 3), with data expressed as mean ± standard deviation (SD). IC_50_ values obtained from the DPPH and ABTS•⁺ radical scavenging assays and EC_50_ values obtained from the FRAP assay were compared between Md-AgNPs and the ascorbic acid (AA) standard using an unpaired Welch's t-test performed in GraphPad Prism 9. Differences were considered statistically significant at p < 0.05.

**Anticancer Studies**

The cells were procured from National Center for Cell Science (NCCS), Pune, Maharashtra, India. The cells were cultured in DMEM culture medium with 10% fetal bovine serum (FBS) and 1% penicillin-streptomycin and incubated at 37°C in a humidified atmosphere with 5% Co_2_. Cells were plated at a density of [X × 10^4^ cells/well] in 96-well culture plates and allowed to attach for 24h (MTT cytotoxicity assay). The sample was prepared as a stock solution and serially diluted with complete culture medium to give the required concentrations. Then cells were treated for 24h with different concentrations (5-100 µg/mL) of the sample. The untreated cells were used as the control.

After the treatment period, 10µL MTT solution (5mg/mL) was added to each well and incubated at 37 °C for 4h. After incubation the medium was discarded and the formazan crystals were dissolved in 100µL dimethyl sulfoxide (DMSO). The plate was then gently shaken to make sure the formazan crystals were completely dissolved and the absorbance read at 570 nm with a microplate reader. Each treatment concentration was done in triplicate and the experiment was repeated three times independently. Data are presented as mean ± SD of three independent experiments. Cell viability is expressed as a percentage of the untreated control (defined as 100%). The IC_50_ value represents the concentration of the test sample needed to reduce cell viability by 50%. It was determined using nonlinear regression analysis in a dose response model in GraphPad Prism. Statistical significance between experimental groups was determined by one-way analysis of variance (ANOVA).

**Cell morphology analysis**

The human breast cancer cells (MCF-7) were grown on cover slips (1 x 10^5^ cells/cover slip) incubated for 24 h with the various concentrations (10 µg/mL, 25 µg/mL and 50 µg/mL) of the Md-AgNPS. They were then fixed in an ethanol:acetic acid solution (3:1; v/v). The cover slips were gently mounted on glass slides for the morphometric analysis. Three monolayers per experimental group were photo micrographed. The morphological changes of the selected MCF-7 cells were analyzed using Nikon (Japan) bright field inverted light microscopy at 40x magnification.

**Figure S1. Photographic image of dried *M. domestica* peel: Inset: Image of fresh apple**

**Figure S2. HPLC profile of *M. domestica* peel extract**

**Figure S3. Photographic images of antibacterial activities and zone of inhibition of Md-AgNPs against (a) *S. aureus*; (b) *S. pyogenes*; (c) *A. hydrophila* and (d) *E. coli* bacteria**

**Figure S4.** The synthesized Md-AgNPs and doxorubicin inhibit MCF-7 cells proliferation in a dose dependent manner for 24 h, the cell viability was determined and the results were expressed as percentage cell viability with control. Results shown are mean, which are three separate experiments performed in triplicate (n=3). *p*-values were calculated using a one-way ANOVA and significance levels were denoted as * *p* < 0.05; ** *p* < 0.01; *** *p* < 0.001.

**Figure S5.** The synthesized Md-AgNPs and doxorubicin inhibit HUVEC cells proliferation in a dose dependent manner for 24 h, the cell viability was determined and the results were expressed as percentage cell viability with control. Results shown are mean, which are three separate experiments performed in triplicate (n=3).

**Table S1** Minimum inhibitory concentration (MIC) and turbidity for different concentrations of samples after 24 h.

| **Sample code** | **Test pathogen** | **250 µg/mL** | **100 µg/mL** | **50 µg/mL** | **25 µg/mL** |
| --- | --- | --- | --- | --- | --- |
| **Md-AgNPs** | *S. aureus* | _ | _ | _ | + |
|  | *S. pyogenes* | _ | _ | _ | + |
|  | A. hydrophila | _ | _ | _ | _ |
|  | E. coli | _ | _ | _ | + |
| **Md-extract** | *S. aureus* | _ | _ | _ | + |
|  | *S. pyogenes* | _ | _ | + | + |
|  | A. hydrophila | _ | _ | _ | + |
|  | E. coli | _ | _ | _ | + |
| **AgNO_3_** | *S. aureus* | _ | _ | _ | + |
|  | *S. pyogenes* | _ | _ | _ | + |
|  | A. hydrophila | _ | _ | _ | _ |
|  | E. coli | _ | _ | _ | _ |
| **Gentamicin** | *S. aureus* | _ | _ | _ | _ |
|  | *S. pyogenes* | _ | _ | _ | _ |
|  | A. hydrophila | _ | _ | _ | _ |
|  | E. coli | _ | _ | _ | _ |

Positive (+): Turbidity indicating growth; Negative (–): No turbidity indicating absence of growth in broth

**Table S2** Minimum Bactericidal Concentrations (MBC) of samples after 24 h

| **Sample code** | **Test pathogen** | **250 µg/mL** | **100 µg/mL** | **50 µg/mL** | **25 µg/mL** |
| --- | --- | --- | --- | --- | --- |
| **Md-AgNPs** | *S. aureus* | _ | _ | + | N/A |
|  | *S. pyogenes* | _ | _ | + | N/A |
|  | A. hydrophila | _ | _ | _ | N/A |
|  | E. coli | _ | _ | + | N/A |
| **Md-extract** | *S. aureus* | _ | _ | + | N/A |
|  | *S. pyogenes* | _ | _ | N/A | N/A |
|  | A. hydrophila | _ | _ | + | N/A |
|  | E. coli | _ | _ | + | N/A |
| **AgNO_3_** | *S. aureus* | _ | _ | + | N/A |
|  | *S. pyogenes* | _ | _ | + | N/A |
|  | A. hydrophila | _ | _ | _ | N/A |
|  | E. coli | _ | _ | _ | N/A |
| **Gentamicin** | *S. aureus* | _ | _ | _ | _ |
|  | *S. pyogenes* | _ | _ | _ | _ |
|  | A. hydrophila | _ | _ | _ | _ |
|  | E. coli | _ | _ | _ | _ |

Positive (+): Turbidity indicating growth; Negative (–): No turbidity indicating absence of growth on agar. N/A – concentration was not considered for testing on agar.

**References**

S1 Alshameri AW, Abdualgalil B, Mutahar M (2025) Green biogenic fabrication of AgNPs derived from *Pulicaria jaubertii* leaf extract: Investigation of antimicrobial and cytotoxic properties. OpenNano 24:100248-100263. [10.1016/j.onano.2025.100248](https://doi.org/10.1016/j.onano.2025.100248)
S2 Shih CL, Jau TL, Chin KW, Hsin YC, Deng JY (2010) Antioxidant properties of various solvent extracts from lychee (*Litchi chinensis Sonn*.) pericarp. Food Chem 114:577-581. <10.1016/j.foodchem.2008.09.088>
S3 Re R, Pellegrini N, Proteggente A, Pannala A, Yang M, Rice-Evans C (1999) Antioxidant activity applying an improved ABTS radical cation decolorization assay. Free Radic Biol Med 26:1231–1237. <https://doi.org/10.1016/S0891-5849(98)00315-3>
S4 Benzie IF, Strain JJ (1996) The ferric reducing ability of plasma (FRAP) as a measure of "antioxidant power": the FRAP assay, Anal Biochem 239:70-76. <https://doi.org/10.1006/ABIO.1996.0292>
S5 Tamuly C, Hazarika M, Bora J, Gajurel PR (2014) Antioxidant activities and phenolic content of Piper wallichii (Miq.) Hand.-Mazz. Int J Food Prop 17(2):309–320. <https://doi.org/10.1080/10942912.2011.631250>
